# Photonic online learning: a perspective

**DOI:** 10.1515/nanoph-2022-0553

**Published:** 2023-01-09

**Authors:** Sonia Mary Buckley, Alexander N. Tait, Adam N. McCaughan, Bhavin J. Shastri

**Affiliations:** Applied Physics Division, National Institute of Standards and Technology, Boulder, CO 80305, USA; Department of Physics, Engineering Physics and Astronomy, Queen’s University, Kingston, ON, Canada

**Keywords:** integrated photonics, neural networks, neuromorphic photonics

## Abstract

Emerging neuromorphic hardware promises to solve certain problems faster and with higher energy efficiency than traditional computing by using physical processes that take place at the device level as the computational primitives in neural networks. While initial results in photonic neuromorphic hardware are very promising, such hardware requires programming or “training” that is often power-hungry and time-consuming. In this article, we examine the online learning paradigm, where the machinery for training is built deeply into the hardware itself. We argue that some form of online learning will be necessary if photonic neuromorphic hardware is to achieve its true potential.

## 1 Introduction

Neuromorphic engineering aims to implement neural networks in hardware by combining neurophysiological principles with engineered device physics [1]. Neuromorphic hardware could break the limitation of conventional digital computers in terms of speed and energy efficiency [2] for implementing artificial intelligence (AI) applications enabled by machine learning. A wide variety of devices have been proposed for this new paradigm, including analog, digital, and hybrid analog–digital CMOS technology [3, 4], memristive devices [5], magnetic tunnel junctions [6], superconducting devices [7], and indeed a variety of photonic platforms. Neuromorphic photonics [8]—combining photonic device physics with distributed processing models—has resulted in a new class of ultrafast information processors [9]. Free space optical neural networks were first demonstrated in the 1980s and 1990s [10–12] while more recent neuromorphic photonic demonstrations range from free-space [13, 14] to integrated [15, 16] implementations, spiking neural networks [17–19] and artificial and deep neural networks [20, 21], to reservoir computing [22–24]. However, the training of neuromorphic hardware is still primarily performed on conventional computers. Referred to as offline learning, in this paradigm, network parameters (e.g., weights and biases) are determined in software based on a computational model of the physical system, and then these parameters are mapped to the physical device which is used for inference. Offline learning has proven to be a valuable tool in neuromorphic engineering, well-suited to mass production, where the results of a single simulation can be mapped to large numbers of devices. For offline learning to be effective, very accurate models of the individual network devices must be developed. One significant body of ongoing work in photonic neuromorphic engineering is optimizing the reliability of the design, fabrication, and manufacture of optical devices such that offline training leads to reproducible results. However, offline training may rule out various devices that cannot be easily modeled and simulated in this way – for example analog devices, or non-standard architectures such as highly recurrent or nonlinear networks. Offline learning is also very power-hungry for applications that often need retraining, resulting in a static model that is hard to adapt to new data and adjust for different scenarios [25]. An alternative approach is needed for these cases. Online learning may be just this alternative, allowing new classes of devices and architectures to be developed and providing other new capabilities that cannot be provided with offline training alone. In fact, many large-scale neuromorphic demonstrations in both photonics [13, 26], [27], [28] and other platforms [29–32] have leveraged some form of online learning.

Online learning refers to training that takes place on the same hardware that is used for inference. Online learning can be either supervised or unsupervised; the critical feature is that it is done without requiring an external model of the device being trained. This article will focus on supervised learning, as it is the most common type of training used on photonic hardware. While reliable digital platforms such as very large-scale integration (VLSI) devices may be effectively simulated and do not necessarily require online learning [33], online learning is already a topic of significant investigation in analog VLSI systems [34], memristive crossbar arrays [29, 30, 35, 36] and a variety of other novel architectures with complex, recurrent or nonlinear interactions that cannot be easily modeled or described mathematically [32, 37], [38], [39]. At the device level, variability can lead to degradation in the measured inference accuracy compared to the expected, offline-simulated inference accuracy. This is an endemic, historically significant issue for many types of emerging hardware. In Section 2, we will discuss how recent approaches have addressed variability for photonic devices. Ultimately, many of these offline training solutions require sacrificing some of the inference capabilities of the device to make it more “trainable” or adding models that include individual device data to the training simulation. At the system level, new architectures could have significant computational power [40] if a feasible training technique could be identified, and nonlinear effects and crosstalk could be harnessed rather than compensated. In Section 3, we discuss the experimental progress on implementing different online training techniques and how they have already enabled improved performance at certain tasks.

From a hardware perspective, the complexity of the devices used for online learning will need to be increased compared to inference-only devices. The exact nature of these new devices and components will depend on the specific learning algorithm and the degree of autonomy required. However, this complexity would also bring a great deal of flexibility to these systems; allowing them to compensate for variability and noise, ultimately leading to much larger networks and enabling us to take full advantage of its information processing capabilities. In Section 4, we make the argument that photonics, in particular, is a good candidate for online learning and that many successful experimental demonstrations of photonic neuromorphic systems, from the 1980s to the present day, have involved some form of online training. Although the problem of training optical neuromorphic hardware has been considered since the 1980s [11] and likely earlier, a one size fits all solution has not been found. The further development of online algorithms and the associated physical implementations will greatly enable the scaling of photonic neuromorphic systems and enhance their performance. Online learning could allow photonic neuromorphic systems to entirely avoid issues with imperfect modeling and thermal and electrical cross-talk, and could help foster the next generation of photonic neuromorphic systems that are competitive with digital electronic systems at AI tasks.

## 2 Training: from offline to online

### 2.1 Offline training

Training for most machine learning systems is done with an optimization technique known as gradient descent, using the backpropagation algorithm to calculate the gradient [41, 42]. The idea is that a large quantity of “training data” is fed into the network, while the network’s output is compared to the known, desired output, and an error or cost is computed. The gradient of the weights and biases with respect to the cost is calculated, and the weights are adjusted in the opposite direction to this gradient. When training on hardware for machine learning, the most common technique is to map the weights and biases from a computer-modeled network trained in-silico using gradient descent via backpropagation to an equivalent hardware network. This is “offline” training, and it works very well for digital systems in which all device characteristics are known with high accuracy. While it is possible to perform other types of training and map them to hardware, in practice, offline training is almost always accomplished via backpropagation. There are many advantages to this type of training. With offline training, hardware can leverage the latest software techniques/computational power for training neural networks. One training simulation can be used to program the weights and biases on multiple, identical devices – assuming those devices do not have too much variability. The weight update process in hardware does not need to be fast, as the weights are programmed into the system and do not need to be modified regularly.

However, there are also some significant disadvantages to offline training. Many emerging hardware platforms, particularly analog hardware, suffer from deviations from the original design parameters. These imperfections can significantly degrade the effectiveness of offline training, resulting in an inference accuracy that will ultimately be much lower than the simulated model accuracy without further optimization or measurements. This is due to the fact that in a deep network or general hierarchical process, even a few per cent difference between the simulated model and reality can result in large inaccuracies. Ref. [43] describes this for a very simple hierarchical model, where a 0.5% change in one of the model parameters can lead to a 30% difference in the output after 20 layers. Additionally, it is often difficult or impossible to accurately model effects such as noise or cross-talk for offline-training. Even when these effects are small, they can distort the in-silico training process and significantly reduce the inference accuracy obtained in hardware compared to the expected modeled accuracy.

Training on models that account for hardware considerations and imperfections is often known as “hardware-aware” training. Although hardware-aware training improves accuracy and can improve other parameters such as static and dynamic power consumption [44], it typically makes the simulations more complicated, cumbersome, and slow. The measurement and evaluation of individual devices needed to build more hardware-aware models can also be involved and may limit ultimate device scaling. For example, in crossbar arrays with ReRAM devices, an analog memory technology being heavily investigated in both industry and academia, it is still typical for the training simulation to include a physical model of the device and to monitor and re-adjust conductance values of the devices during programming to improve performance [45]. In optical devices, similar hardware-aware approaches are common, with compensation both for the behavior of individual devices and device cross-talk between devices [46, 47], which adds significantly to time and energy costs of training.

Another approach to mitigate the device-to-device variability is to model and operate the hardware as a low-bit-depth digital system, where the bit depth is chosen such that the system can be modeled accurately despite device-to-device variations. This is common in other neuromorphic hardware platforms, such as memristive crossbar arrays [48] or phase change materials [35], where bit depths of 6–8 can be achieved by careful device engineering and characterization. In photonic systems, achieving high bit-depth typically means using feedback and measurement mechanisms to improve the stability of the weights, increasing the overall effective bit depth and therefore the ease of mapping the model to hardware [49]. Bit-depth can also be increased by implementing the weights in a time-multiplexed digital configuration [50]. These solutions increase overhead in time, experimental complexity, or device size. Alternatively, some researchers have focused on developing training techniques that work with extremely low bit-depth, even limiting themselves in some cases to single bit [51]. In either case, even though inference tasks can, in principle, be done with analog representations of numerical values, the hardware is instead operated in a digital configuration so that the trained model can be more easily mapped to the hardware.

### 2.2 Autonomous online learning

One potential solution to many of these issues is to build hardware that can train itself, in a process that we refer to here as “autonomous online learning”. In this type of self-training device, also referred to as a physical learning machine [39], the training data and labels would be fed directly into the device, and it would adjust its weights and biases autonomously in response. No computer would be required in any part of the process, and therefore there would be no need to bring the chip offline for training, as it could be retrained in the field. Online training would allow training of fully analog systems, with fabrication imperfections accounted for during the training, obviating the need for making numerous measurements to characterize individual components. This is possible in online training because the machinery for compensating for the non-idealities is built into the device. Autonomous online learning has been implemented in several digital hardware platforms as a means to reduce the energy cost of training [25, 52], and great progress has been made in circuits for autonomous online learning in analog or analog–digital platforms such as platforms based on analog CMOS [36], memristive crossbar arrays [35], and indeed photonics [28, 53, 54].

Truly autonomous learning has yet to be demonstrated in photonics. This is due to the complexity of including all of the components needed for training in hardware. Implementing autonomous online learning is particularly onerous in the case of the backpropagation algorithm as it requires hardware implementation of backward communication channels, baked-in knowledge of nonlinear derivatives, and more. Because of this, although there have been proposals for performing backpropagation on free space [53, 55], [56], [57], [58] and integrated photonic hardware [54, 59], many other online training proposals suggest altering the training algorithm to make it more hardware-friendly [28, 60] (see Section 3). However, no matter what algorithm is used, one difficulty in photonic hardware is that fast weight updates are needed, as weights are always dynamically updated during online training. Thermal tuning with ring resonators [15, 61] or Mach–Zehnder interferometers [20] (MZIs) is one of the main techniques for implementing weights in integrated photonic hardware. While simple to implement with heaters, thermo-optic tuning can be slow (kHz), power-hungry (often requiring milliwatts of constant power draw per weight), and their stabilization can be challenging; however, with feedback control techniques, record weight precision of 7 bits [62] and recently, 9 bits [49] have been reported. The high-power consumption of thermal tuning is an issue for offline and online training and will require further advances. While other options exist for fast (GHz) and energy-efficient tuning, including carrier injection (forward-bias PIN junctions) or carrier depletion (reverse-biased PN junctions), their limited tuning range means that the ring resonators need to be supplemented with post-fabrication trimming techniques [63]. In contrast, carrier effects do not typically achieve the index changes necessary to operate MZI neural networks. Other potential technologies being investigated for weight tuning include micro- and nano-electromechanical effects [64], piezo-optomechanical effects [65], and optical phase change materials [66, 67], which all promise faster tuning with lower static power consumption, but present new materials and integration challenges.

### 2.3 Hardware-in-the-loop

**Figure 1:**
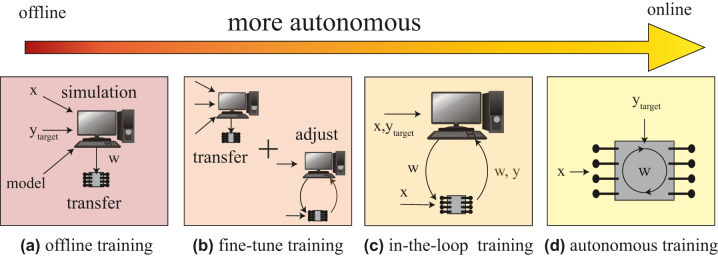
Training on photonic hardware ranging from fully offline to fully online. (a) Offline training, where a model of the physical system and the training dataset are trained on a computer, and the weights are transferred to the device. (b) “Fine-tune training”, where the system is trained as in (a), but the weights are adjusted to improve the accuracy after transfer to the device. (c) Hardware-in-the-loop involves measuring the chip during training, but some portion of the calculations for training still happen on a computer. (d) Fully autonomous online learning. In this case, only the training dataset is input to the device, which can adjust its weights autonomously as it learns.

In practice, training does not need to be either fully offline or online; instead, there may be a continuum between offline and online autonomous training. This is shown in Figure 1. One approach often used is “hardware-in-the-loop” or “chip-in-the-loop” training. Rather than internal computations performing self-adjustments during training, the device parameters will be read out externally to a computer that computes the necessary weight updates. This is done in a “loop” such that the weights are dynamically updated during the training. Hardware-in-the-loop algorithms may require offline computations that use some or all of (i) the output of forward pass (inferences) read from the device during training, (ii) weights dynamically read from the device during training, and (iii) a physical simulation of the device. The fewer these elements required, the closer the training becomes to being “autonomous online”. The on-chip part of the calculations used in hardware-in-the-loop can compensate for fabrication imperfections or differences between model and reality. Depending on the algorithm used, it is possible to avoid building a hardware simulation model. Therefore chip-in-the-loop is more suited to analog systems than offline training. As with online training, fast weight updates are needed, as weights are dynamically updated during training. Since all of the weights must be updated in a loop, there may be I/O issues causing speed bottlenecks during this type of training, especially as the system is scaled up. Like online training, hardware-in-the-loop may also benefit from algorithms other than backpropagation. In practice, due to the complexity of electronic-photonic integration, most of the online training that has been demonstrated so far has been some form of hardware-in-the-loop training.

**Figure 2:**
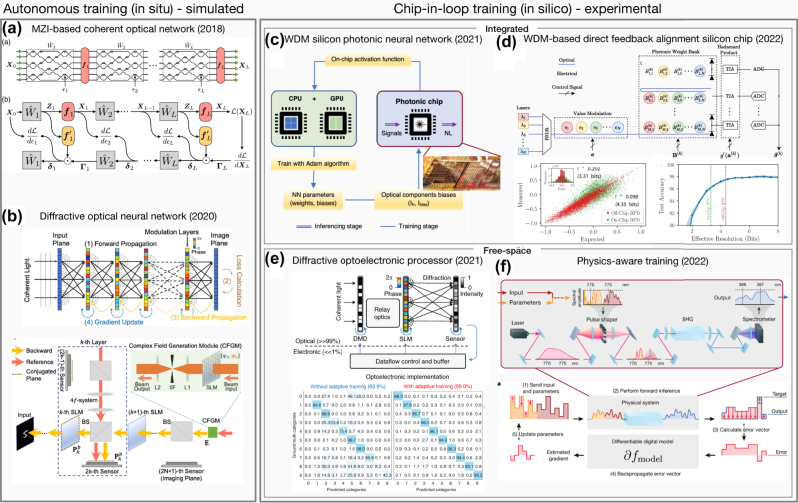
Proposals for training photonic hardware. (a) and (b) Simulations of fully autonomous online learning proposals for gradient descent via backpropagation in (a) integrated photonics [59] and (b) free space [56]. (c)–(f) Experimentally demonstrated hardware-in-the-loop. (c) The measured activation functions are used in the hardware-aware training of micro-ring resonator based neuromorphic device for optical signal processing [61]. (d) Integrated implementation of DFA where the backward pass is computed on-chip [60]. (e) Fine-tune training of the network increases the accuracy of handwritten digit recognition from 63.9% to 96% [68]. (f) Training of a free-space physical neural network where the calculation of the forward pass in the device prevents accumulation of errors that can happen in offline training [43].

### 2.4 On-chip “fine-tune” training

In between chip-in-the-loop and offline training is another case that we call “fine-tune” training, which is also sometimes referred to as adaptive training. In this case, a “coarse” training is initially performed in-silico via backpropagation on a simulated network, and the trained parameters are programmed to the photonic device. After weights are programmed, the device’s performance is evaluated, and the weights may be updated via either further simulation or hardware-in-the-loop type updates. Fine-tune training can combine some of the advantages of offline training with the benefits of online training. Fine-tune training can reach a higher accuracy than just offline training [68] because the on-chip fine-tuning compensates for defects/variations. It can also be used to reduce the energy cost of the trained model, for example, by setting certain inconsequential voltages to zero and repeating training. For hardware with slow weight updates, fine-tune training could help to account for fabrication variability without requiring as many dynamic weight changes. The major disadvantage is that it requires multiple training techniques, which can add overhead to training. The more fine tuning needed, the more the speed of the weight change matters (e.g., thermal tuning will be very slow). The more measurements that need to be made, the further away from being truly “offline” that it is. The best training approach will generally depend on the hardware and the application. However, online training could have many advantages if it is truly realized in photonic hardware.

### 2.5 Other approaches

While these considerations apply to most photonic neural networks, it is worth mentioning a few special cases for which some of the above discussions do not apply. Spiking neural networks are a class of neuromorphic networks that try to emulate how information is encoded in the brain. Many photonic hardware platforms have been proposed using spiking optical signals [18, 69, 70]. However, the brain’s algorithm for learning with spiking signals is not well understood. There are many techniques for encoding information in spiking signals, each requiring special treatment. Learning in spiking systems is beyond the scope of this article, but a detailed introduction to the issues and challenges can be found in Ref. [71].

Reservoir computing is another proposed type of computing with many potential photonic implementations [23, 24, 72, 73]. The idea with reservoir computing is not to train on the hardware but instead to take a complicated, linearly inseparable problem and, using hardware, perform a complex nonlinear transformation to turn it into a problem that can be solved more easily in software (for example with a linear solve), or another simpler hardware device at the input/output of the reservoir [73, 74]. Reservoir computing is simpler than many other network types to implement in photonics because it does not require hardware training. Despite the relative ease of implementation of reservoir computing, current theory cannot connect reservoir characteristics – such as size, network topology and nonlinearities – to computational performance. Therefore, its potential for reliably solving larger-scale problems in realistic contexts has yet to be demonstrated. In the case of reservoir computing, online learning for the internal reservoir weights likely will not apply.

## 3 Experimental progress in online training of photonic hardware

### 3.1 Backpropagation in hardware

Backpropagation is an algorithm for calculating the gradient of the cost with respect to network parameters for gradient descent optimization [41]. Backpropagation followed by gradient descent is the most commonly used technique for training deep neural networks in software and therefore has been an obvious choice for direct implementation in hardware for online training. Although hardware for implementing backpropagation is still in the development phase in most hardware platforms, there has been significant progress in fully analog networks where circuits for weight updates have been demonstrated [36], and memristive crossbar arrays [35], where the relevant circuits have been simulated and evaluated.

The backpropagation algorithm involves computing the derivatives of the cost with respect to the outputs of neurons in the last network layer, and then recursively computing loss gradients in previous layers using the chain rule. The chain rule computation requires a layer-wise multiplication of the transpose of the weight matrix by the error signal. This part of the backpropagation calculation is very well suited to optical implementations: most photonic neural network backpropagation schemes leverage the fact that optical synapses or weights are bi-directional [55]. A common hardware implementation uses a conjugate mirror to reflect an optical error signal through the same optical path as in the forward direction, thus propagating it through the transpose of the weight matrix in the forward direction, a technique that was first employed in the 1980s in free space optical networks [10, 53]. The major difficulty is that this symmetry provides only one part of the backpropagation computation – it does not account for the nonlinearity, which must be implemented differently in the forward and backward processes. Proposals have typically approached this by using a different optical frequency or power in the backward direction and choosing a material or device with an optical nonlinearity that behaves differently in this configuration, such as Fabry–Perot etalons [53] or saturable absorbers [57]. Another proposal [75] is to use a specific activation function with a constant derivative. Alternatively, Hughes et al. [59] propose computing the required function digitally and applying it to the backpropagating signal electro-optically. Some experimental proposals for backpropagation in hardware are shown in Figure 2.

A fully autonomous online implementation of backpropagation in photonic hardware has not yet been realized experimentally due to the challenging nature of the proposed experiments. In an integrated demonstration, Pai et al. [54] implemented the error computation proposed in Ref. [59] experimentally in an MZI vector-matrix multiplier by implementing beam taps to read off the forward and error signal and perform the necessary computations for the weight adjustments in a hardware-in-the-loop configuration. Similarly, the proposal of Wagner et al. [53] was partially implemented experimentally, with the requisite nonlinearity calculation computed on an external computer. The success of these hardware-in-the-loop demonstrations shows the importance of adding an online training to photonic neuromorphic hardware. Despite this progress, these proposals are all significantly more challenging than inference-only operation. These difficulties are due to the properties of the backpropagation algorithm, which has motivated the investigation of more hardware-friendly algorithmic approaches.

In an alternative to online error computation, several papers [43, 76] showed that inference accuracy could be improved by implementing only the forward pass in hardware and computing the errors and nonlinear activations in software in another variation of a hardware-in-the-loop technique. The advantage of this technique is that differences between model and experiment are partially compensated for in each pass, as there is experimental feedback. Even though a model of the system is needed for training, the model accuracy may not need to be as good due to this forward-pass compensation. It is important that, going forward, the effectiveness of these techniques are evaluated for large-scale networks, where, for example, approximations made in the backward direction may have more of a detrimental effect. Many techniques that work on small, shallow networks can break down on the larger deep neural networks used to classify modern datasets, so investigation at scale is crucial [77, 78].

### 3.2 Approximate gradient descent in hardware

An important point to note is that backpropagation is not the optimization algorithm used on neural networks but rather just a technique used to calculate the gradient. In fact, there are alternative methods for calculation or approximation of the gradient. One of the conceptually most straightforward methods is the finite difference. In finite difference, every weight in the network is perturbed by a small amount, Δ*w*, and the change in the cost, Δ*C*, is recorded. The ratio Δ*C*/Δ*w* is used as an approximation for the partial derivatives ∂*C*/∂*w*, which are combined to construct the gradient and perform a step in gradient descent. As the perturbation size goes to zero, the gradient approximation becomes exact. A hardware implementation was demonstrated in Ref. [20] as a method for training an MZI optical neural network. However, the disadvantage of this technique is that it requires perturbing every parameter before taking a step, keeping track of the order of perturbation globally, and, if implemented on-chip, it requires an extra per-synapse memory to keep track of each gradient component. Together, these issues have kept finite difference an unappealing technique.

These challenges can be overcome in hardware by modifying the gradient approximation technique, for example, by perturbing parameters simultaneously and performing updates more frequently, as in the simultaneous perturbation stochastic approximation (SPSA) algorithm [80]. SPSA significantly speeds up the training process when compared to finite-difference, and eliminates the need for individual memories at each parameter to store the gradient component. Additionally, the parameter perturbations can be random and either analog or discrete, making the technique asynchronous and flexible for different types of weight implementations. SPSA was first implemented in analog VLSI hardware in the 1990s [81, 82] on small-scale problems such as 2 bit parity and has even been shown to be effective on recurrent neural networks [83]. More recently, there has been a resurgence of interest in this type of training on emerging analog neuromorphic hardware platforms, from memristive crossbars [84] to a recent demonstration on photonic MZI neural networks with integrated nonlinearities [28] solving a vowel recognition problem. Many different perturbative algorithms, including SPSA, can be implemented on-chip or as a hardware-in-the-loop process using a hardware-friendly framework called multiplexed gradient descent MGD. It has recently been shown that using MGD with realistic hardware parameters, MGD can be competitive with backpropagation in terms of speed and accuracy as it is scaled up to large problems [85].

**Figure 3:**
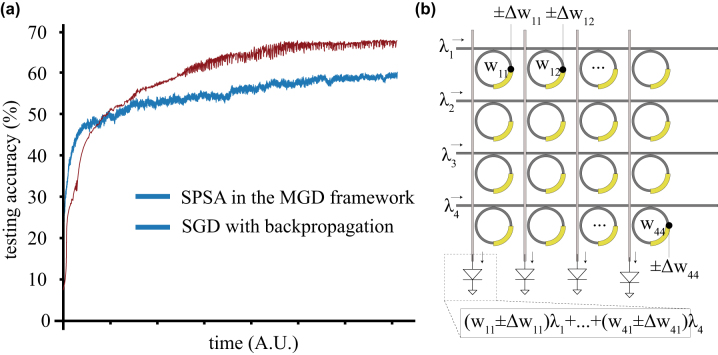
(a) CIFAR10 testing accuracy versus time during training with SPSA in the MGD framework, as compared to SGD with backpropagation. The time axis has been scaled for MGD operating with 20 kHz weight perturbations, compared to wallclock time for backpropagation performed on a standard desktop GPU. (b) Schematic illustration of perturbing the weights in a microring resonator implementation of photonic neuromorphic hardware. The implementation is shown in a two-layer waveguide process that allows simple waveguide crossings [79] for illustrative purposes; the network can also be realized in a single waveguide layer as in ref. [15].

Figure 3(a) shows a comparison of the SPSA algorithm (as implemented in MGD [85]) to backpropagation, training on the CIFAR-10 dataset in a network with ≈30,000 weights and biases. The MGD simulations have indicated that, for realistic hardware parameters (for example, hardware in which the weights can be perturbed at kHz-MHz speeds), it could be competitive in training speed with backpropagation on a standard GPU for the CIFAR10 and FashionMNIST image recognition datasets. Additionally, tests of this framework have shown that it is robust to realistic types of noise and defects present in photonic neural networks such as fabrication imperfections and analog noise. As a result, these techniques avoid many of the pitfalls of implementing backpropagation on hardware – they can be implemented on existing inference-only chips and are robust against practical defects. Shown in Figure 3(b) is a schematic demonstration of how MGD could be implemented in a microring resonator based photonic neural network by adding a small, discrete perturbation to the weights on MRRs. These perturbations generate a corresponding change in the cost that can be used for the training signal. Since MGD does not require a model of the system or knowledge of architecture, activation functions or activation function derivatives, the same approach will work on physical neural networks, recurrent networks, and a variety of photonic neural networks, including MZI networks, superconducting opto-electronic networks, and even other, more exotic network types.

Another technique proposed in Ref. [86] is feedback alignment. Feedback alignment replaces the weight matrix transpose used for backpropagation with a random matrix while keeping the other elements of the backpropagation algorithm the same. Despite this no longer being a good calculation for the gradient, it turns out that the system will still move toward decreasing cost due to the over-defined nature of most neural networks. Furthermore, an even simpler follow-up to this technique, called direct feedback alignment (DFA), was proposed in Ref. [87]. Instead of propagating the error signal layer by layer via the chain rule, the error from the last layer is used everywhere in the network. DFA has been shown empirically to be effective in some networks. Optically, this is appealing, as a single random scattering medium can implement the large, random weight matrix needed. This has been demonstrated in free space optics to train on the MNIST dataset in a hardware-in-the-loop configuration with the optics performing the random matrix multiplication [27]. There has also been work towards implementing DFA in an integrated platform with on-chip microring resonator weight banks [60], in which the algorithm was simulated with realistic hardware parameters. Like SPSA, since DFA does not require the chain rule step of backpropagation or feedback alignment, it can also be applied to physical or recurrent neural networks, or as feedback to a reservoir, as was demonstrated in Ref. [74]. The main issue with DFA is that while it has been shown to work for smaller, shallow networks, there is evidence that neither feedback alignment nor DFA generalizes to larger, deeper networks [77, 78]. However, modifications to the feedback alignment algorithm appear to ameliorate this issue [88], although, in practice, these may prove more challenging to implement in hardware. Figure 2 shows some experimental progress towards implementing these types of approximate gradient descent training techniques in photonic hardware.

Other approaches for approximating gradient information have also been proposed. These include subspace descent [89] and others. It seems likely that these types of algorithms will become more and more prevalent as new machine learning hardware develops. All this research is relatively new, and our understanding of these algorithms will likely continue to evolve and improve.

### 3.3 Other training techniques

Gradient descent is not the only optimization technique used for training neural networks. In particular, genetic algorithms and evolutionary optimization have proved effective for training recurrent neural networks that perform time-dependent control functions. Typically, these algorithms rely on a global reward parameter for training and do not require large, labeled datasets, which can appeal to many applications in which training data is unavailable. An example of a free space optical implementation of a genetic algorithm used to solve a control problem was shown in ref. [90]. There have also been proposals for evolutionary optimization of photonic spiking networks [91]. These algorithms can be used to train the network architecture and parameters. However, they will be tricky to implement in online learning, as the training rules often involve evaluating multiple configurations and generating diverse network candidates that are subsequently evaluated and pruned. Evolutionary and genetic algorithms will likely be essential for spiking and recurrent networks, but more experimental investigations are needed.

A particularly effective approach for control tasks is the class of algorithms known as reinforcement learning. Like evolutionary and genetic algorithms, reinforcement learning only requires a global reward function rather than large, labeled training datasets. In Ref. [13], Bueno et al. demonstrated a free space version of an optical reinforcement learning algorithm. Reinforcement learning may be a good candidate for online training, and although many different versions exist, much research remains to determine the best option for photonic hardware.

Another important class of training algorithms is energy-based, in that the system dynamically settles to some minimum energy, and this minimum energy in some way contributes to solving the problem. These have been extensively explored for hardware implementations, as energy minimization is a natural phenomenon in physical systems. If it can be appropriately harnessed, the physics of the system could be used to solve machine learning problems. Examples of energy-based training algorithms include the training of Hopfield networks [92], Boltzmann machines [93] and, more recently, equilibrium propagation [94].

Early optical demonstrations of energy-based optical networks included free-space holographic [95, 96] and optoelectronic [97] implementations, although these were used in relatively small networks. More recently, energy-based systems have been attracting renewed interest across many different hardware platforms, with examples of recent demonstrations including a Boltzmann machine comprised of magnetic tunnel junctions that autonomously learns by contrastive divergence [6], as well as a recent demonstration of autonomous training by equilibrium propagation in a table-top resistive network [32]. There has also been a resurgence of work on optical Ising machines [98, 99] using the physics of coupled optical parametric oscillators (OPOs) to perform the necessary energy minimization. These OPOs have been scaled up to systems of hundreds of thousands of optical spins [100] and have already surpassed CPUs at specific problems [101]. It is notable that this class of algorithms and training share similarities with adiabatic quantum computing which is currently viewed as a promising pathway for solving computational problems using near-term, noisy intermediate scale quantum devices. Work to use these to solve interesting machine learning problems is ongoing.

There is also a class of biologically inspired, local, unsupervised algorithms based on Hebbian learning rules that we have not discussed in this article. For a review that includes examples of such learning, see Ref. [102].

## 4 The future of training photonic hardware

The development of photonic neuromorphic hardware is still an active research area with many potential paths forward. However, the development of photonic hardware must go hand-in-hand with the development of novel training techniques. The exact training techniques employed will depend on both the hardware and the application, and there will likely not be one single strategy. Currently, most photonic hardware is trained offline, which will remain an important technique. There is significant ongoing research to address current issues and improve the quality of offline training. However, in addition to offline training, we will see more and more of the training process being implemented in the hardware, i.e., online. Ultimately, full utilization of hardware compute capabilities will require some degree of online training to provide a way to account for hardware non-idealities, allow analog operation, and for the training of systems with novel device physics that may not be easily simulated in software. New online training techniques will span a range from “fine-tune training” to fully autonomous online training.

The scaling of photonic hardware is another ongoing challenge that must be addressed with new training techniques. For offline training, device-to-device variability poses more significant challenges at scale. At the same time, many new algorithms and techniques being developed for more efficient training also fail for larger-scale networks. A truly scalable online training technique will likely need to be implemented autonomously to overcome I/O challenges associated with in-the-loop techniques. However, in the near term, the experimental challenges of a fully-autonomous system mean that demonstrations are likely to include some amount of “in-the-loop” character – this is true even for many photonic inference demonstrations, where the vector-matrix multiplications are performed in hardware, but the implementation of the nonlinearity is often performed using traditional digital arithmetic sandwiched between analog-to-digital and digital-to-analog converters.

Experimentally, for online training to be truly viable, electronics and photonics must be integrated into a single system at a low level. This is becoming possible with the recent development of large-scale high-performance commercial joint electronics-photonics processes [103] and commercial platforms with high-speed electrical connections between photonic and electronic chips [104]. Low-power static and dynamic weights must be developed, as current techniques in integrated photonic platforms are power-hungry and too slow for efficient online training. It will be exciting to see the progress in this field and the new advances that spring from research in this direction. We believe that there are many intermediate opportunities for improving existing techniques by adding on-chip elements to the training algorithms. While a photonic neuromorphic device that truly trains itself is perhaps a long-term and high-risk research goal, if realized it could be truly transformational.
